# Ultrasonic Welding of PBT-GF30 (70% Polybutylene Terephthalate + 30% Fiber Glass) and Expanded Polytetrafluoroethylene (e-PTFE)

**DOI:** 10.3390/polym13020298

**Published:** 2021-01-19

**Authors:** Dan Dobrotă, Sergiu Viorel Lazăr

**Affiliations:** 1 Department of Industrial Engineering and Management, Faculty of Engineering, Lucian Blaga University of Sibiu, 550024 Sibiu, Romania; 2 S.C. Contintental Romania, 550018 Sibiu, Romania; sergiu.lazar@ulbsibiu.ro

**Keywords:** ultrasonic welding, PBT-GF30 (70% polybutylene terephthalate + 30% fiber glass), expanded polytetrafluoroethylene (e-PTFE), parameter optimization

## Abstract

The ultrasonic welding of polymeric materials is one of the methods often used in practice. However, each couple of material subjected to ultrasonic welding is characterized by different values of technological parameters. Therefore, the main objective of the research presented in this paper is to optimize the parameters for the ultrasonic welding of two materials, namely PBT-GF30 (70% polybutylene terephthalate + 30% fiber glass) and expanded polytetrafluoroethylene (e-PTFE). In this sense, the research was carried out considering a plate-type part made of PBT-GF30, which had a thickness of 2.1 mm, and a membrane-type part made of e-PTFE, with a thickness of 0.3 mm. The condition imposed on the welded joints made, namely to correspond from a technical point of view, was that the detachment pressure of the membrane should be at least 4 bar. To this end, a test device was designed. Additionally, the topography of the material layer from the plate-type part was analyzed, as well as the chemical composition and surface condition for the membrane-type part. The obtained results allowed the optimization of the following parameters: The welding force; welding time; amplitude; and holding time. All experimental results were processed using STATISTICS software, which established how each parameter influences the characteristics of welded joints.

## 1. Introduction

Currently, for an increasing number of parts made of composite materials and used in various industries, attempts are being made to eliminate the classic ways of joining them (mechanical joints and the use of adhesives) [1,2,3]. The best known welding techniques available for composite bonding are resistance [4], induction [5], and ultrasonic welding [6]. In the case of ultrasonic welding, the effect of different welding parameters, such as the welding time, welding pressure, ultrasonic vibration amplitude, holding time, and holding pressure, on the welding quality was previously investigated. It was found that the amplitude of ultrasonic vibrations and geometry of the energy director (ED) had a very large influence on the quality of welded joints made with ultrasound [7].

The possibilities of ultrasonic welding of composite materials made of fiberglass reinforced with polypropylene (PP) and composite materials of fiberglass reinforced with nylon have been investigated. It has been shown that the ED geometry has a significant effect on the quality of the weld because it allows energy concentration during the joining process. It was shown that the semicircular shape is the most efficient welding condition, while the triangular ED displayed the lowest result. It has also been shown that during ultrasonic welding, the parts must be tightened in a controllable manner. At the same time, it has been demonstrated that the use of an ED, which has a similar geometry to the welded product, results in a considerable improvement of the welded joints. Under these conditions, proper design of the ED results in a considerable improvement in the strength of the welded joint, which can be explained by the optimal transformation of ultrasonic energy into heat [8,9,10].

Ultrasonic welding is a very efficient process, especially in the case of welding polymeric materials, due to the fact that it does not cause degradation of their properties. Crystalline thermoplastic materials are also considered to have the best performance in ultrasonic welding [11].

To achieve joints by ultrasonic welding, the use of a frequency in the range of 18–70 kHz is recommended, depending on the characteristics of the materials to be welded. Moreover, these ultrasonic vibrations must have a small amplitude (i.e., 30 to 100 µm peak-to-peak) and, at the same time, with the introduction of ultrasonic vibrations, a constant static pressure must be applied. The interface generates heat by surface friction and viscoelastic heating. Continuous ultrasonic welding (CUW) has been shown to be a fast and feasible welding technique. The feasibility of CUW was demonstrated when joining polyphenylene sulfide (CF/PPS) plates reinforced with 100 mm carbon fiber. However, it has been found that the joints thus obtained are patchily welded, and this may influence the joints of parts that have certain sealing conditions [12].

A particular problem that occurs with ultrasonic welding is that the maximum thickness of the welded materials is limited. This is due to the fact that vibration penetrates parts made of thicker materials with difficulty and ultrasonic vibration in the joint area is thus not able to produce quality welding [13]. Therefore, at present, the welding thickness is limited to about 3 mm due to the strength of the equipment used to weld [14].

During use of the ultrasonic welding process, some of properties of materials have negative effects on the quality of welded joints. For example, properties such as a high rigidity, hardness, and damping factor negatively influence the quality of the welded joint, because their high values prevent the transformation of ultrasonic energy into thermal energy [15,16]. Furthermore, a major disadvantage of ultrasonic welded joints is related to the fact that the parts obtained by this welding technology have a reduced resistance to fatigue due to the cyclic vibrational load that occurs during welding [17,18,19].

Ultrasonic welding (USW) is a promising method for the welding of dissimilar materials. Ultrasonic thermal welding by the third phase (TWTP) method was proposed in combination with the formation of a third phase, which was confirmed to be an effective technology for the polymer welding of two dissimilar materials compared with the traditional USW [20,21]. Research on the effect of the orientation of PBT-GF30 fibers on the strength of ultrasound welded joints has been conducted. It was established that, when fibers are oriented parallel to the mold surface, cracks were detected at the welded surfaces, leading to a reduction in the vibration welded (VW) joint strength [22].

Other welding technologies such as laser welding technology (LW) were used to weld the parts made of PBT-GF30, but it was found that in order to obtain a proper joint, it is necessary to place a layer of polycarbonate between parts subjected to welding (PC) [23].

From an analysis of the current state of research, it can be concluded that the application of ultrasound is very often used for welding thermoplastic materials. However, research in the field of the welding of thermoplastic materials with reactive processing, such as polyurethanes (PU), polybutylene terephthalate (PBT), and acrylic resin, is very limited. Therefore, the main objective of the research presented in this paper is to optimize the parameters for the ultrasonic welding of two materials, namely PBT-GF30 (70% polybutylene terephthalate + 30% fiber glass) and expanded polytetrafluoroethylene (e-PTFE).

## 2. Materials and Methods

### 2.1. Materials

The ultrasonic welding assembly process was performed considering two components: A plate-type component, made of PBT-GF30 (70% polybutylene terephthalate + 30% fiber glass), and a membrane component, made of expanded polytetrafluoroethylene (e-PTFE). By assembling these types of parts, the aim was to create a product that must have a tightness at a pressure of 4 bar.

#### 2.1.1. Properties of PBT-GF30 Material

The housing-type part was made of PBT-GF30 material (70% polybutylene terephthalate + 30% fiber glass); had a thickness of 2.1 mm; and was supplied by Julier (Xiamen) Technology Co., Ltd., Fujian, China.

PBT-GF30 is a thermoplastic, semi-crystalline plastic of the polyester family, which crystallizes very slowly and is therefore in an amorphous-transparent or crystalline-opaque state, depending on the processing method. It is distinguished by its high strength, rigidity, and dimensional stability under heat, as well as by its very high dimensional stability and low creep. In addition, PBT shows, like polyesters in general, very good friction and wear properties. PBT has a good impact resistance, especially in the cold. PBT GF30 has optimized properties in different areas compared to PBT. The properties of PBT GF30 are a high strength and rigidity, high dimensional stability, very high dimensional stability, low creep, very good friction and wear resistance, good impact resistance, very low thermal expansion, good chemical resistance to acids, very good electrical properties, very low water absorption, and the way in which it can be easily bonded and welded.

Regarding the distribution of the fiber length in such a composite material, it can be established considering the weighted average fiber length (Lw). The properties of fiberglass are presented in Table 1.

**Figure 1:**
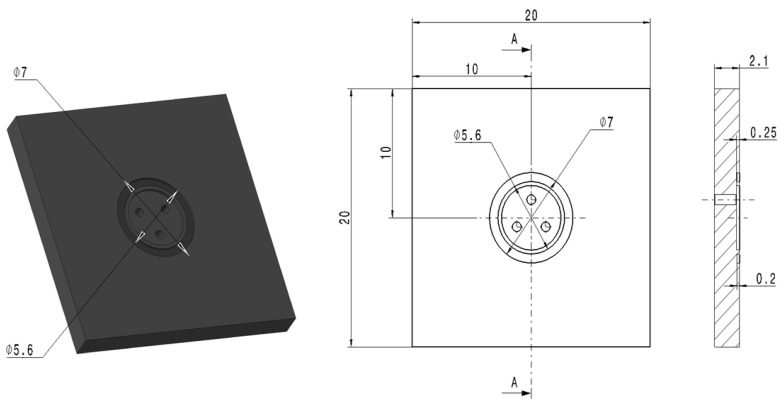
The shape and dimensions of the plate-type part.

From the PBT-GF30 material, a plate-type part was made, in which three holes of ϕ 0.75 mm through which fluids could penetrate were provided; these fluids can have a maximum pressure of 4 bar. A sketch of this type of part is shown in Figure 1.

#### 2.1.2. Properties of the e-PTFE Material

The membrane-type component was made of expanded polytetrafluoroethylene (e-PTFE); had a thickness of 0.3 ± 0.05 mm; and was supplied by W. L. Gore & Associates, Inc., Elkton; Maryland, USA. Additionally, the membrane used in the research was in the shape of a square, with a side dimension of 8 ± 0.05 mm. The choice of this type of membrane was made considering the fact that it has multiple uses due to its properties, such as its ability to achieve an incredible tightness; ability to seal damaged flange surfaces, resistance to creep and cold flow, superior blowout and high temperature performance, longer service life (typically without a need for retorque), and superior reliability performance. This type of membrane was supplied in rolls with the following dimensions: Outer diameter of 250 mm and inner diameter of 76.5 mm. It should also be noted that this type of membrane allows the passage of water in one direction.

### 2.2. Methods

#### 2.2.1. Realization of the Welded Joint with the Help of Ultrasound

The process of forming an ultrasonic welded joint of polymeric matrix composites is particularly complex and can be conventionally divided into three stages:

- In the first stage, there is perfect cleaning due to the phenomenon of acoustic cavitation, which occurs as a result of the propagation of ultrasonic waves;
- In the second stage, the ultrasonic vibrations cause the development of heat on the surfaces in contact due to the existence of relative movement between them, with ultrasonic frequency. The heat resulting from the friction of contact surfaces causes most of materials to melt in a very short period of time;
- In the third stage, connections appear between contact surfaces heated up to the plastic state temperature, allowing the realization of a welded joint with a good resistance.

Parts subjected to the ultrasonic welding process must not be contaminated (dust, grease, moisture, mold release agent, etc.) or mechanically damaged. In this sense, they were degreased and cleaned. A semi-automatic ultrasonic welding system produced by SONIC ITALIA SRL, Viale de Gasperi, 20017, Rho, Milan, Italy, was used to perform ultrasonic welding. This system allows the adjustment of sonotrode frequencies to the values of 20, 30, 35, and 40 kHz, respectively, and amplitude values from 5 to 50 μm. During the experimental research, a sonotrode frequency of 35 kHz was employed, and the other parameters of the welding process had different values, according to the data presented in Table 2.

From an analysis of the data presented in Table 2, it was observed that in order to achieve optimization of the parameters of the ultrasonic welding process, different values needed to be taken into account for three of the parameters, namely, the welding time, welding force, and amplitude. These parameters were chosen because they influence the properties of ultrasonic welded joints the most significantly.

**Figure 2:**
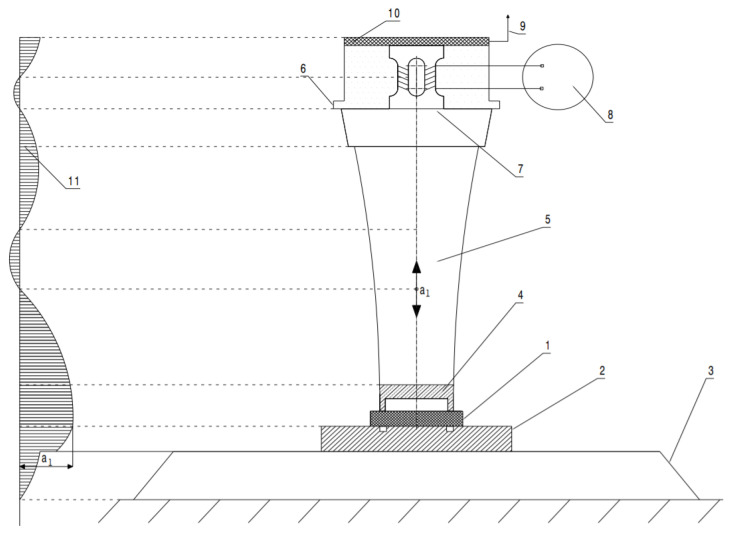
Schematic diagram for welding materials: 1—membrane-type part; 2—plate-type part; 3—acoustic anvil; 4—sonotrode; 5—ultrasonic energy concentrator; 6—nodal flange; 7—ultrasonic transducer; 8—ultrasound generator; 9—connection element to the power source; 10—acoustic insulating; and 11—diagram of variation of the amplitude of the particle velocity throughout the ultraacoustic system.

The scheme of the ultrasonic welding technology is presented in Figure 2. It should be specified that the variant in which the direction of the pressing force coincides with the direction of longitudinal oscillations was chosen.

As can be seen in the diagram in Figure 2, welding in the “near field” or contact welding with an ultrasonic approach was used to make the welded joint, where the sonotrode was brought as close as possible to the joint area. In this case, the ultrasonic energy was evenly distributed over the entire contact surface of parts 1 and 2, which were welded. The front part of sonotrode 4, which came into contact with the upper part, had the same surface and shape as the parts to be welded.

#### 2.2.2. Topography of the Surface Layer of the Plate-Type Part

In order to obtain a very good welded joint, it is important to perform an analysis of the topography of the surface layer of the parts subjected to the welding process. In the case of the plate-type part, the emphasis was placed on identifying the variations of the dimensions because it has a more complex shape, but also on establishing the roughness of the surface of the part in the joint area. The analyses were performed with a Dimension Edge AFM system (Bruker, Billerica, MA, USA).

#### 2.2.3. Scanning Electron Microscopy (SEM) Analysis of the Membrane-Type Part and Welded Joint

Obtaining a welded joint to ensure a very good tightness is possible in conditions in which there is a good adhesion between the membrane-type part and plate-type part. In this sense, an SEM analysis was performed for both the surface of the membrane part and the surface obtained in the joining process. Therefore, information about the topography of the membrane surface, the joining surface of parts, and the chemical composition of the membrane-type part was obtained. An AIS2100C electron microscope produced by SERON TECHNOLOGIES INC., 5F World Vision Bldg., 209, Gyeongsu-daero, Uiwang-si, Gyeonggi-do, Korea, was used in the research.

#### 2.2.4. Testing the Welded Joint from a Tightness Point of View

**Figure 3:**
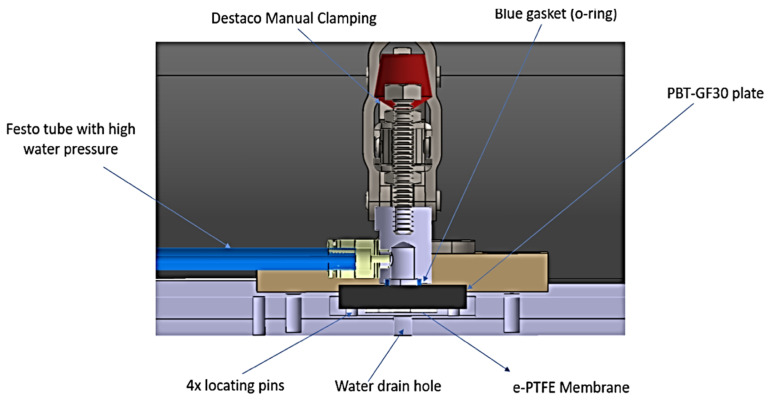

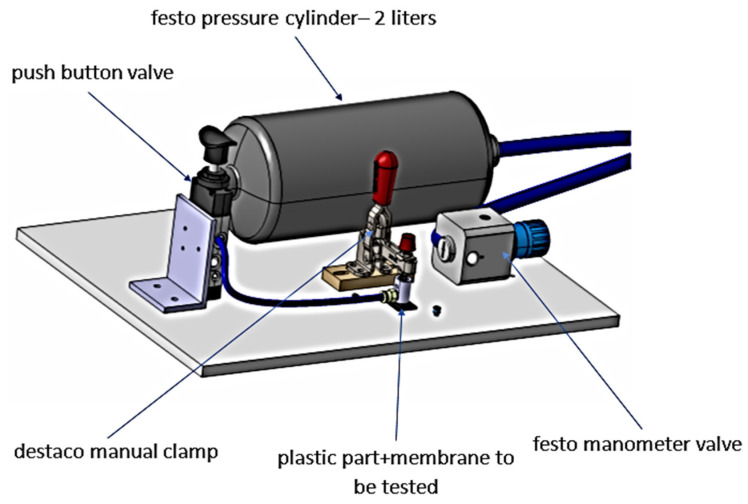
Scheme of the device used to test welded joints in terms of tightness.

The welded joint made between the plate-type part and membrane-type part was tested from the point of view of the tightness. For this purpose, the device shown in Figure 3 was used. The water pressure test was performed at a maximum pressure of 5 bar, and the water temperature was 23 °C ± 2 °C. The water pressure in the installation was gradually increased, and in case tightness of the joint at pressures above 4 bar was not ensured, it was considered that the welded joint was not appropriate.

## 3. Results and Discussion

### 3.1. Results Obtained When Measuring the Surface Topography of the Plate-Type Part

**Figure 4:**
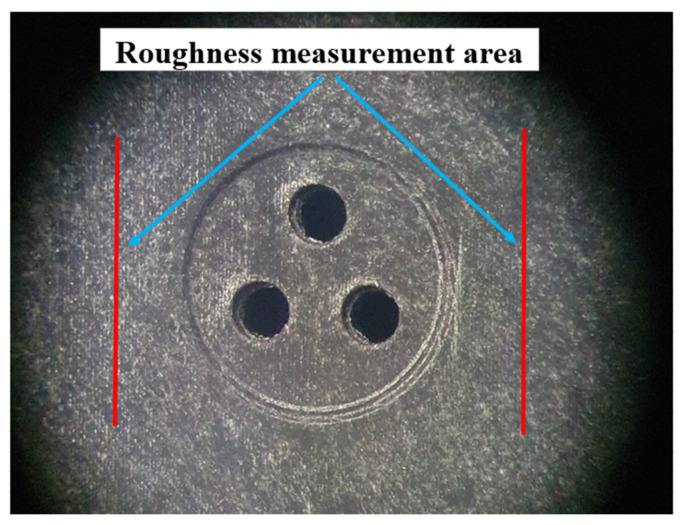
Roughness measurement area.

**Figure 5:**
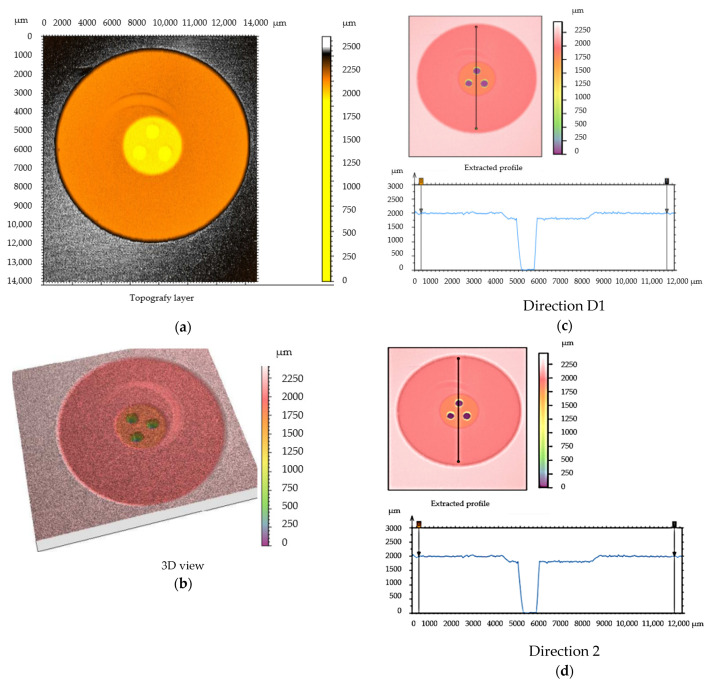
Analysis of the topography of the plate-type part. (**a**)–topography layer; (**b**)–3D view; (**c**)—thickness variation on D1 direction; (**d**)—thickness variation on D2 direction.

The topography of the shape of the housing-type part is of particular importance for any deviations from the dimensions. High values of roughness for the surface of a part can also cause changes in the characteristics of the welded joint. In this sense, it was decided that the roughness should have values in the range of Ra = 2–4 µm. For measuring the surface roughness and determining the wall thickness for the plate-type part, 10 samples were analyzed. In the stage of measuring the surface roughness of the plate-type part, a measuring area was established, as shown in Figure 4. Additionally, the topography of the part’s surface was recorded in two distinct directions, as presented in Figure 5. The measurement of these parameters was performed according to the methodology presented in Section 2.2.2.

The results obtained after an analysis of the surface topography of the plate-type part for the 10 samples are presented in Table 3.

From an analysis of the data presented in Table 3, it was observed that there are some differences in terms of the surface roughness, but also in terms of the thickness, for the plate-type parts analyzed. The thickness of the plate-type part exhibited differences in thickness, ranging from a minimum value of −0.96 µm to a maximum value of 2.45 µm. Differences in thickness were recorded in both directions of measurement, and their presence was determined by the manufacturing technology of the part. All these differences can influence the operating performance of welded joints.

This topography of the work part surface has a special practical influence because each microneregularity of the surface is a concentrator of acoustic energy and the first melting areas will appear in the microneregularities of the highest height. The molten material is expelled into the micro-recesses of the lower surface, contributing to acceleration of the melting process of the other microneregularities, which is a process intensified by the ultrasonic energy introduced in the area to be joined.

Research has shown that the higher the micro-irregularities of the contact surfaces, the faster the welding process and the better quality the joint that is obtained [24,25,26]. These influences can be explained by the fact that the process of forming the ultrasonic welded joint can be conventionally divided into two stages:

- In the first stage, ultrasonic oscillations cause the development of heat on contact micro-irregularities between the two surfaces. These micro-irregularities move relative to each other, with an ultrasonic frequency and a certain amplitude, resulting in a large amount of heat due to contact friction. Most thermoplastics start to melt in a very short time;
- In the second stage, when heated up to the temperature of the plastic state, there are connections between the contact surfaces that allow a resistant joint to be obtained, after all the microneregularities have melted, creating a homogeneous area on the entire contact surface.

The temperature in the joint area must be lower than the minimum temperature at which, under the given conditions, destruction of the material in the contact area can occur, and higher than the temperature at which a resistant joint can be obtained. In order to analyze the influence of the plate topography on the performance of welded joints, the 10 samples with the characteristics presented in Table 3 were combined. Given that the parts to be welded are part of a large series of processes, it is very important to manufacture as many parts as possible in the shortest period of time. Therefore, in the first phase, the use of a welding time that was as short as possible was proposed. The 10 samples were thus ultrasonically welded considering a holding time of 150 ms. The resulting energy was determined based on data obtained from the equipment used for welding. The welding energy directly depends on the amplitude of vibrations, which must be correlated with the welding time and welding forces, but also on the roughness of the surfaces of the welded parts. Considering this, the research aimed to optimize three parameters, namely, the amplitude, welding forces, and welding time, because their optimal values will result in the optimal energy result. After establishing several preliminary parameters of the welding process, 10 samples were obtained, as shown Table 4, which were subjected to the pressure test using the device shown in Figure 3. All of the tests performed showed that none of the samples corresponded to the imposed pressure conditions, with the obtained values for the detachment pressure of the membrane-type part being less than 1 bar.

The results presented in Table 4 demonstrate that, if the surface roughness is higher, a welded joint with superior characteristics can be obtained, as demonstrated by sample 9. It was also observed that, if the plate-type part is a little thinner, sample 9 causes a rise in temperature in the welding area, with positive effects on the characteristics of the welded joints. In addition, none of the welded joints made met the condition that the detachment of the membrane-type part be constructed at a pressure of 4 bar. The detachment of the membrane for these joining conditions by welding was performed at low water pressure values below 1 bar, well below the minimum allowed limit of 4 bar. The observed pressure differences can be explained by the fact that the samples had different roughnesses.

**Figure 6:**
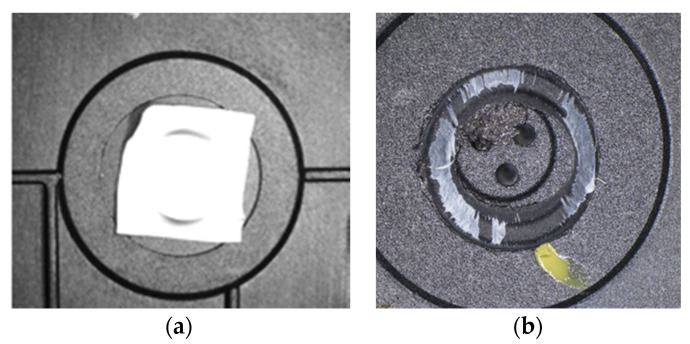
Sample 1 obtained by ultrasonic welding. (**a**)—initial condition and (**b**)—after detaching the membrane-type part.

Therefore, even though the welding looked relatively good from the outside, as shown in Figure 6a, the pressure tests failed. The main problem identified in ultrasonic welding based on the principle of high productivity is that welding is not completely performed. The impression left by the sonotrode on the joint surface should be a complete circle, but after the tests were performed, incomplete traces of the circle obtained by welding were observed, presented in Figure 6b.

### 3.2. Results Obtained When Analyzing the Membrane-Type Part

**Figure 7:**
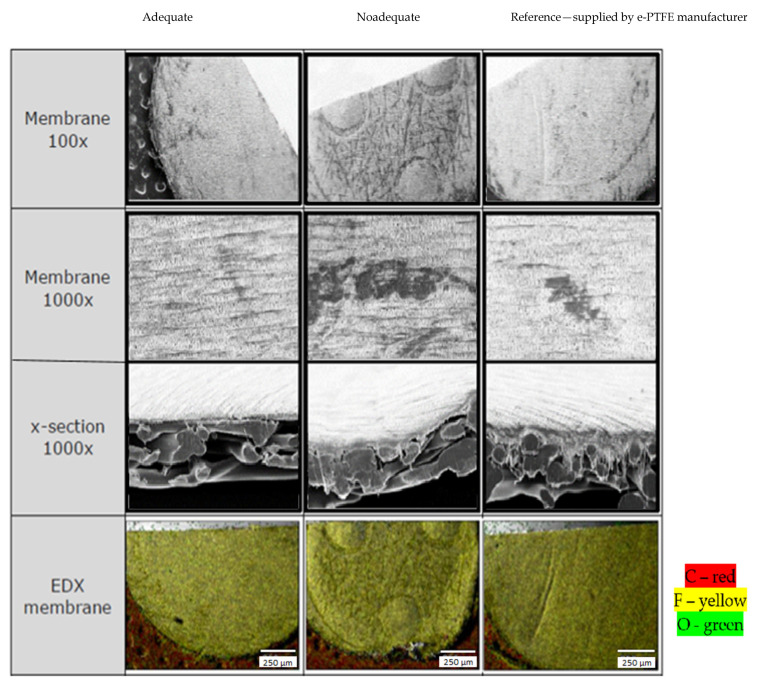
Analysis of the membrane-type part made of expanded polytetrafluoroethylene (e-PTFE).

Obtaining good results of the welded joint using PBT-GF30 and e-PTFE is possible in conditions where the characteristics of the membrane-type part made of e-PTFE are appropriate in terms of the condition of the outer surface of the structure in the section and the chemical composition. In this sense, the membrane-type part was subjected to SEM analysis and the results presented in Figure 7 were obtained.

SEM analysis in the case of the membrane-type part made of e-PTFE showed that, in the case of this type of part, there may be certain defects in the surface layer, but also some deviations of structure compared to the reference. This analysis was performed considering a reference value, depending on which it was established whether the membrane was appropriate. The analysis of the surface layer of the membrane showed that there may be areas of the membrane-type part which are characterized by discontinuities of the surface layer; an aspect that can greatly influence the performance of welded joints. Furthermore, from the analysis of a section of the membrane, it was found that in some areas, gaps may appear in the section, which makes the membrane inadequate. The membrane with the smallest gaps in the section was considered adequate and could be compared with the reference value.

**Figure 8:**
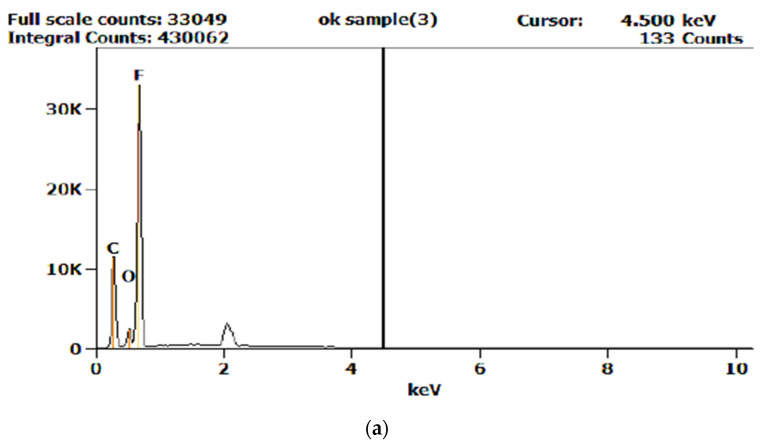

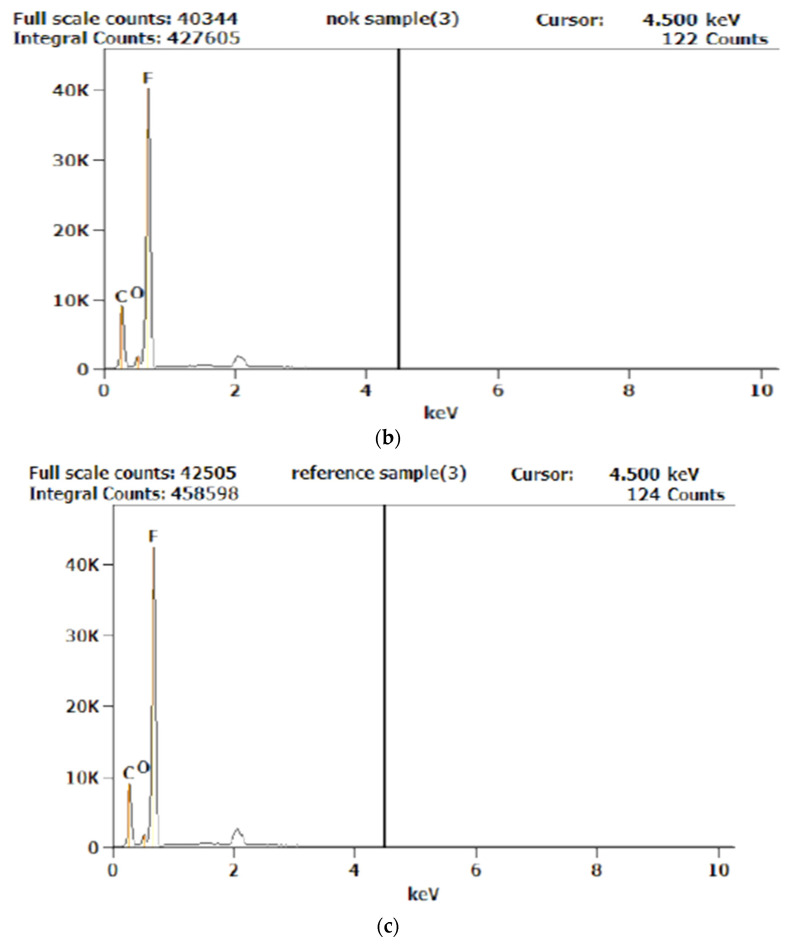
Graphs showing the evolution of the chemical composition of e-PTFE obtained by Energy-dispersive X-ray spectroscopy (EDX) analysis: (**a**) Adequate chemical composition; (**b**) inadequate chemical composition; and (**c**) reference chemical composition (supplied by the e-PTFE manufacturer).

One of the factors that can influence the performance of welded joints is the chemical composition of the membrane. In this sense, the Energy-dispersive X-ray spectroscopy (EDX) method was used to determine the chemical composition of the membrane. Following this analysis, different values of the chemical composition were found, namely for the elements C, O, and F. Employing a reference value for the chemical composition of the membrane, a limit value of ±5% was established, against which the chemical composition could be accepted. The analysis showed that there were differences in the chemical composition for e-PTFE, and the values obtained are shown in Table 5. The analysis of the chemical composition was performed for all membrane-type parts used in the research. Therefore, the values of the determined chemical composition were compared with the values transmitted by the e-PTFE supplier. Parts of the e-PTFE membrane that did not have an adequate chemical composition were not used in the experiments. Moreover, graphs of evolution of the chemical composition obtained from the EDX analysis are shown in Figure 8.

### 3.3. Optimization of Ultrasonic Welding Process Parameters

In order to optimize the parameters that can be adjusted for the ultrasonic welding of PBT-GF30 and e-PTFE, we employed the results obtained in the preliminary research presented in Table 4, which showed that by choosing non-optimized parameters, welded assemblages cannot be obtained, ensuring detachment of the membrane-type part at pressures of at least 4 bar. Detachment of the membrane at pressures lower than 4 bar is explained by the fact that the welding was not completely performed on the entire circumference. Under these conditions, it was necessary to modify the welding parameters so that the membrane was properly welded. In the research from this stage, the same 10 plate-type parts with the characteristics presented in Table 3 were taken into account.

**Figure 9:**
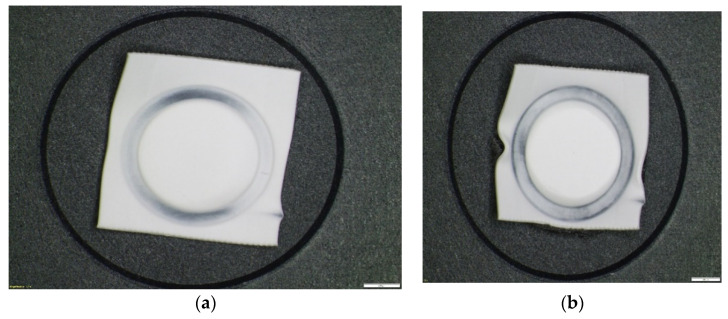
Images of the specimens obtained with different parameters of the ultrasonic welding regime: (**a**) Sample 5 and (**b**) sample 3.

Therefore, for the following samples, new intervals of variation of parameters for the welding regime were established, namely, the welding force had values in the range of 75–95 N, the welding time was increased to a value of 450 ms, the amplitude was decreased to 70%, the holding time had a constant value of 150 ms, and the energy result had correspondingly higher values. Adjusted parameters, but also the results obtained, are presented in Table 6. Moreover, an image of sample 5, which withstood the lowest pressure, is shown in Figure 9a, and an image of sample 3, which withstood the highest pressure, is shown in Figure 9b.

From the analysis of images presented in Figure 9, it can be observed that, in the case of sample 5, which withstood the lowest pressure, there is no interpenetration of the e-PTFE membrane material with the PBT-GF30 plate material, except in a very small area, while in the case of sample 3, interpenetration of the two materials occurs for the entire circumference.

Additionally, the results obtained showed that the size of the pressing force decisively influences the value of the average breaking strength of ultrasonic welded joints. It was also found, however, that the optimum value of force can only be determined when the welding time and amplitude of the sonotrode are taken into account. At the same time, it was found that there is a close connection between the pressing force and the amplitude of the active part of the assembly, in the sense that if there is a pressing force and an amplitude of the active part of the sonotrode, the resistance of the joint decreases substantially, as is the case with sample 5, which has a high force value and low amplitude.

The pressing force at welding also has a special influence on the local static pressure of contact, increasing as the static pressing force increases at different acoustic energy densities. The static pressing force is also chosen, depending on the thickness of the parts to be joined, with there being an optimum for the resistance of the joined material, depending on the thickness and the pressing force.

The duration of the welding, i.e., the duration of action of ultrasonic waves in the contact area, has a special influence, not only on the possibility of making the joint, but also on the quality of the welded joint and the resistance of the joint. The experimental results obtained showed that there is an optimal value for the welding time, depending on the thickness of the material to be welded. Therefore, the maximum resistance of joined material changes according to the thickness of the parts to be joined.

**Figure 10:**
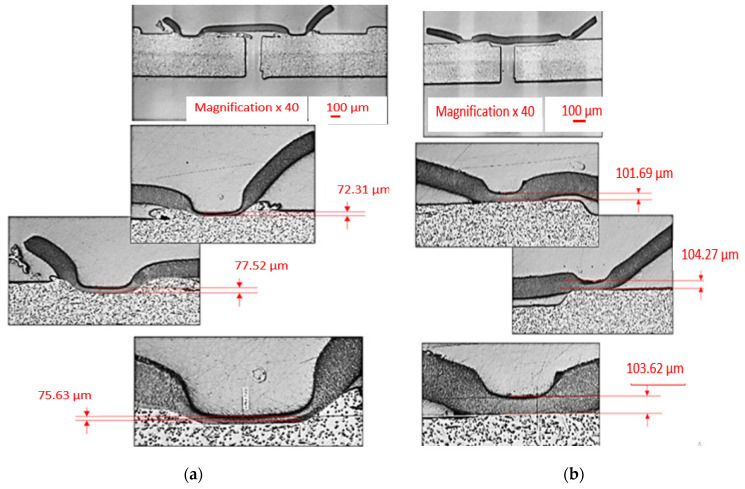
Membrane welding cross section: (**a**) Section through test tubes that have withstood a detachment pressure greater than 4 bar, sample 3, and (**b**) section through samples which have withstood a detachment pressure of less than 4 bar, sample 5.

**Figure 11:**
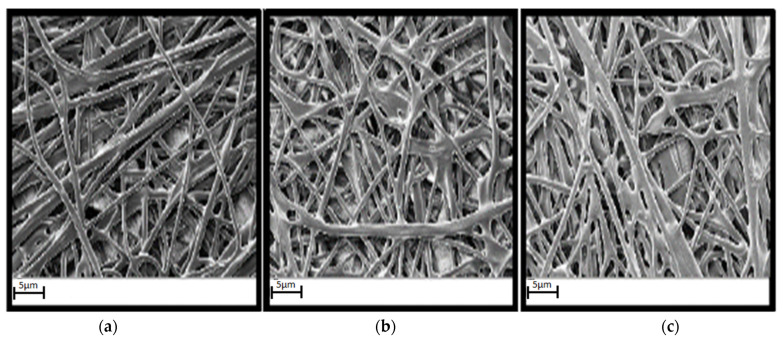
The shape of the surface in the detachment area of the membrane: (**a**) Sample 5; (**b**) sample 8; and (**c**) sample 3.

In order to observe the way in which welded joints were made, a series of samples were investigated with SEM, as shown in Figure 10 and Figure 11. In the first stage, an SEM analysis was performed for two samples, namely, sample 3, as presented in Figure 10a, and sample 5, as shown in Figure 10b. The two samples were chosen because sample 3 withstood the highest detachment pressure of the membrane-type part, and in the case of sample 5, the membrane detachment pressure had the lowest value. Two sections were made through the membrane to observe the ultrasonic welding area. Following this investigation, it was observed that the membrane part had different thicknesses in the joint area, and in the material from the plate-type part, a discharge must occur in the welded joint area to obtain a suitable welded joint (Figure 10a). The measurement of the thickness of the membrane-type part demonstrated that its optimal size in the joint area must be between 72.31 and 77.52 μm, respectively. Under these conditions, it can be concluded that a too-thick membrane remaining after welding demonstrates insufficient interpenetration of the membrane material in the plate material, and if the membrane is too thin, this substantially decreases its mechanical strength. It should also be noted that the thickness of the membrane must be reduced by about four times in order to obtain a welded joint that can withstand pressures greater than 4 bar. This is demonstrated by the fact that, if the membrane type part initially had a thickness of 300 μm, after welding, in the case of sample 4, an average membrane thickness of approximately 75 μm was obtained.

Following SEM analysis of the membrane detachment area, shown in Figure 11, it was found that in the case of sample 5, which withstood the lowest membrane detachment pressure, there was no proper interpenetration between PBT-GF30 and e-PTFE. In order to increase the performances of the welded joint, the degree of interpenetration of the two materials must be increased, and Figure 11b presents an image of the membrane detachment area for sample 8, which withstood a limit pressure of 4 bar, and, in the case of the sample 3, which showed the highest membrane detachment pressure of 5.1 bar, the best interpenetration between PBT-GF30 and e-PTFE was observed.

In the case of sample 5, Figure 11a, it was observed that the material from the membrane-type part did not adequately cover the surface of the plate part and thus the amount of e-PTFE that adhered to the PBT-GF30 was very small. In the case of sample 8, Figure 11b, a better coverage of PBT-GF30 with e-PTFE was observed due to the improvement of the parameters used for welding, and this determined an increase in the pressure the welded joint resisted. The highest amount of e-PTFE that adhered to the fibers in PBT-GF30 was observed in the case of sample 3, which withstood the highest detachment pressure of 5.1 bar.

Furthermore, the experimental results obtained were processed using STATISTICA 7.0 software (Stafsoft, Inc., Tulsa, OK, USA). The purpose of this statistical processing of experimental data was to establish the optimal values for the parameters of the welding process, but also to identify those parameters that have the greatest influence on the characteristics of the welded joint and the pressure at which detachment of the membrane-type part occurs.

To determine the impact of the parameter values (welding force, welding time, and amplitude) on the pressure at which the membrane part detaches, the ANOVA method was used, which is a robust method for determining the contribution of each factor and the significance of the optimization model. The values of the Fischer test (F value), the sum of the squares, and the parameter_beta were determined. P values below 0.05 or 5% were considered statistically significant. The values determined for p, F, and parameter_beta, respectively, for the situation in which the separate influence of each parameter was analyzed are presented in Table 7.

The values obtained by statistical processing of the experimental data demonstrate that all three analyzed parameters have a significant influence on the value of the pressure at which detachment of the membrane-type part occurs. This was highlighted by the fact that *p* = 0.000007–0.00000012, being less than 0.05. However, an important conclusion that can be drawn is that the amplitude parameter has the greatest influence because the value F = 212.7887 is the largest for this parameter. Based on these results, an optimal value was established for the applicability in the range of 28–32 µm.

However, in order to establish how the three parameters influence the membrane detachment pressure, the parameter_beta values for the situation in which the detachment pressure is influenced at the same time by all three parameters of the welding technology were determined by statistical processing. The following values were determined: Welding force_beta = −0.713; welding time_beta = 0.879; and amplitude_beta = 0.741. From the analysis of these values, it was found that the welding force has a negative influence on the detachment pressure of the membrane, in the sense that an increase in the pressing force causes a decrease in pressure. This can be explained by the fact that too much welding force would cause an exaggerated reduction in the thickness of the membrane part in the area of the welded joint. Under these conditions, the welding force would have lower values than those previously analyzed. Moreover, by increasing the welding time and amplitude, the characteristics of the welded joint can be improved, but at the same time, a certain correlation must be established between the values of these parameters in the sense that none of these must display the maximum values.

Following the analysis of the results obtained during this stage of the research, validation of the optimal values for the welding parameters was carried out. In order to obtain a good welding quality, which also ensures the passing of pressure tests, the conclusions and values previously obtained through statistical processing of the experimental data were taken into account. Therefore, a welding force with a value of 70 N lower than those previously used was chosen. The welding time was maintained at 450 ms, and for the amplitude, an optimal value of 21 µm was established, whilst the holding time remained the same (150 ms). All these parameters were used for joining by welding the same 10 types of plate-type parts previously analyzed with the membrane-type parts, and the obtained results are presented in Table 8.

The results obtained in this stage of the research, as presented in Table 8, demonstrate that a proper choice of the parameters of the ultrasonic welding process allows joints that meet the condition that the detachment of the membrane-type part is achieved at minimum pressures of 4 bar to be obtained. All these results demonstrate the fact that the optimization of the welding parameters was achieved according to the technical conditions, but also the economic ones, maintaining the value of its holding time at 150 ms.

The obtained results confirmed that by increasing the amplitude, the dissipated energy can also increase, except for small amplitudes [27]. Under these conditions, a relatively low amplitude of ultrasonic vibrations allowed better heat dissipation in the joint area and joints with appropriate properties. Furthermore, the obtained results confirmed that, in addition to the amplitude of vibrations, the characteristics of the welded joints largely depend on the welding time, as shown in [28]. At the same time, it has been shown that when the welding force is increased, the maximum temperature in the welding area of the parts is reduced and therefore, welded joints with superior characteristics cannot be obtained. This can be explained by the fact that the imposition of a higher pressing force results in faster flattening of the roughness on the surface of the parts and, thus, the peak effect is limited, preventing the polymer from reaching a high temperature. In fact, other research has shown that a lower welding force allows better heating [29]. Despite the fact that in this research we started from a welding force of 120 N, it was demonstrated that a value much lower than this—70 N—allows the best results to be obtained.

## 4. Conclusions

The research presented in this paper aimed to determine the optimal parameters for the ultrasonic welding of two parts made of PBT-GF30 and e-PTFE, in order to improve the adhesion between them and avoid the appearance of porosity that causes a detachment of parts at pressures higher than 4 bar. In order to better understand the complex interactions between the physical phenomena that occur in the ultrasonic welding of these materials, this paper focused on the analysis of several parameters that characterize an ultrasonic welding process. Several important technological results expected were confirmed for the first time by this study:

- The amplitude of vibrations directly acts on the supplied energy and governs the local heating rate. However, in practice, there must be a correlation between the amplitude values and welding, time in order to prevent rapid flattening of the roughness of the parts, which causes a reduction in the overall efficiency of the ultrasonic welding process. The research initially started from a maximum amplitude of 40 μm, and an optimal value for the amplitude of 30 μm was finally obtained. Moreover, an optimal value of the welding time of 450 ms was established, although the research was initially performed with a welding time of 150 ms;
- Too much welding force results in a welded joint with reduced characteristics, as it causes a rapid change in the contact geometry between the surfaces of the energy parts before reaching a high temperature, and this negatively influences the performance of the welded joints. Therefore, an optimal value of the welding force of 70 N was determined, although the initial research was performed with a welding force of 120 N;
- A higher roughness for the plate-type fabric made of PBT-GF30 can positively influence the characteristics of the welded joint, but too high a roughness can cause the appearance of some porosities and thus may weaken the joint made, with negative effects on the pressure at which the two parts detach. On the contrary, a surface with a lower roughness can lead to progressive filling of the interface between parts with a lower porosity.

Therefore, the parametric analysis detailed in this paper determines a deeper understanding of the ultrasonic welding process, which is essential for any further optimization of the processing parameters. However, in order to determine exactly how to make the welded joint between the two types of materials, further research is still needed. First, the final cooling phase must be investigated. Then, the results obtained need to be validated and improved in order to provide more accurate results. The next step is clearly to extend the analysis to a three-dimensional range to simulate the continuous ultrasonic welding process. It should also be noted that this type of ultrasonic joint has a special applicability in the vehicle industry, where the joints must be solid, airtight, and corrosion resistant. Additionally, these properties of joints made of PBT-GF30 and e-PTFE have not yet been investigated in enough depth and other analyzes need to be performed, considering the long-term behavior of the structures made of such materials at normal loads (creep, high frequency loads, impact loads, etc.). These problems prevent the regular industrial use of the fastest and most easily automated joining technologies, such as ultrasonic welding, for such materials. Future research will also focus on the analysis of possibilities of applying the results obtained for other types of materials that have different properties and greater thicknesses.

## Figures and Tables

**Table 1:** Properties of the glass fibers.

Density (g/cc)	Tensile Strength (GPa)	Poisson’s Ratio	Coefficient of Thermal Expansion (107 K^−1^)	Specific Heat (J/kg.K)	Thermal Conductivity (W/m.K)	Weighted Average Fiber Length (μm)
2.57	2.01	0.23	5.1 × 10^−6^	805	1.35	327

**Table 2:** Ultrasonic welding process parameters.

Parameter	Value
outer diameter sonotrode	7.0 mm
inner diameter sonotrode	5.6 mm
welding time	0.15–0.45 s
hold time	0.15 s
trigger force	0.95 × welding force
welding force	70–120 N
amplitude	30–40 μm

**Table 3:** Results obtained after analyzing the surface topography of the plate-type part.

Samples	Roughness Measurement Results, Ra (µm)	Height Differences Direction 1, µm	Height Differences Direction 2, µm
1	2.32	−0.05	−0.83
2	2.48	−0.15	−0,.3
3	2.07	−0.37	−0.28
4	1.41	0.59	0.63
5	2.07	2.01	2.45
6	2.44	1.26	1.07
7	2.58	−0.59	−0.83
8	2.01	−0.18	0.13
9	2.68	−0.96	−0.98
10	2.23	−0.12	−0.37

**Table 4:** Results obtained after testing welded joints made using parts with characteristics presented in Table 3**.**

Samples	Welding Force, N	Welding Time, ms	Amplitude, µm	Holding Time, ms	Energy Result, J	Membrane Detachment Pressure, bar
1	87	150	40	150	20	0.87
2	87	150	40	150	21	0.91
3	100	150	40	150	20	0.85
4	100	150	40	150	20	0.78
5	110	150	40	150	22	0.86
6	110	150	40	150	22	0.89
7	120	150	40	150	24	0.94
8	120	150	40	150	23	0.84
9	120	150	40	150	24	0.98
10	120	150	40	150	24	0.88

**Table 5:** Chemical composition of e-PTFE.

Qualifying	Element	Net Counts	Weight, %	Atom, %	Compound, %
Not adequate	C	60,736	30.8	40.7	30.8
	O	21,923	9.4	9.4	9.4
	F	195,570	59.7	49.9	59.7
Adequate	C	48,162	26.7	36.1	26.7
	O	18,019	7.3	7.4	7.3
	F	236,610	66.0	56.4	66.0
Reference	C	48,354	26.1	35.5	26.1
	O	18,435	7.1	7.3	7.1
	F	251,100	66.7	57.3	66.7

**Table 6:** Parameters and results obtained in the ultrasonic welding process of PBT-GF30 (70% polybutylene terephthalate + 30% fiber glass) and e-PTFE.

Samples	Welding Force, N	Welding Time, ms	Amplitude, µm	Holding Time, ms	Energy Result, J	Membrane Detachment Pressure, bar
1	85	350	34	150	50	4.2
2	90	300	36	150	39	2.9
3	85	350	36	150	57	5.1
4	80	450	28	150	50	4.3
5	80	450	26	150	39	2.3
6	85	450	28	150	41	4.4
7	75	450	28	150	48	4.5
8	75	450	28	150	48	4
9	75	450	28	150	48	4
10	75	450	28	150	47	4.3

**Table 7:** The values of p, Fischer test (F), and parameter_beta, respectively, after processing the experimental data using ANOVA.

Parameters	*p*	F	Parameter_Beta
Welding force	0.00000012	191.1677	−0.713
Welding time	0.00000009	176.7996	0.879
Amplitude	0.00000007	212.7887	0.741

**Table 8:** Results obtained with the optimal values of ultrasonic welding technology.

Samples	Welding Force, N	Welding Time, ms	Amplitude, µm	Holding Time, ms	Energy Result, J	Membrane Detachment Pressure, bar
1	70	450	30	150	34	4.4
2	70	450	30	150	33	4.1
3	70	450	30	150	35	4.5
4	70	450	30	150	31	4.1
5	70	450	30	150	33	4.2
6	70	450	30	150	31	4.2
7	70	450	30	150	32	4.3
8	70	450	30	150	34	4.2
9	70	450	30	150	31	4.1
10	70	450	30	150	33	4.3
